# A novel knotless hand-sewn end-to-end anastomosis using V-loc barbed suture vs. stapled anastomosis in laparoscopic left colonic surgery: A propensity scoring match analysis

**DOI:** 10.3389/fsurg.2022.963597

**Published:** 2022-11-02

**Authors:** Shining Xu, Xuan Zhao, Zirui He, Xiao Yang, Junjun Ma, Feng Dong, Lu Zang, Abe Fingerhut, Luyang Zhang, Minhua Zheng

**Affiliations:** ^1^ Department of General Surgery, Ruijin Hospital, Shanghai Jiao Tong University School of Medicine, Shanghai, China; ^2^ Shanghai Minimally Invasive Surgery Center, Ruijin Hospital, Shanghai Jiao Tong University School of Medicine, Shanghai, China; ^3^ Section for Surgical Research and Department of General Surgery, Medical University of Graz, Graz, Austria

**Keywords:** barbed suture, laparoscopic surgery, left hemicolectomy, colon cancer, left colon anastomosis, extracorporeal anastomosis, intracorporeal anastomosis

## Abstract

**Background:**

Laparoscopic colectomy is widely practiced for colon cancer, but many variations exist for anastomosis after laparoscopic colon cancer radical resection.

**Method:**

We retrospectively analyzed 226 patients who underwent laparoscopic-assisted radical resection for left colon cancer with knotless hand-sewn end-to-end anastomosis (KHEA) technique with barbed V-loc™ suture material and compared perioperative outcomes, safety, and efficacy to those undergoing stapled anastomosis from 2010 to 2021.

**Results:**

After the 1:2 propensity score matching, 123 participants with similar preoperative characteristics (age, body mass index, TNM stage, and tumor location) were enrolled in the study: 41 in the KHEA and 82 in the stapler group. Statistically significant differences were found in time to accomplish the anastomosis (mean 7.9 vs. 11.9 min, *p* < 0.001) and hospital costs (mean 46,569.71 vs. 50,915.35 CNY, *p* < 0.05) that differed between the KHEA and stapler group, respectively. No statistically significant difference was found in the mean delay to bowel function recovery (2.6 vs. 2.7 days, *p* = 0.466), duration of hospital stay (8.6 vs. 7.9 days, *p* = 0.407), or rate of postoperative complications (14.6% vs. 11.0%, *p* = 0.563). Anastomotic leakage occurred in 11 patients: 5 (12.2%) vs. 6 (7.3%) (*p* > 0.05) in the KHEA and stapler group, respectively.

**Conclusion:**

KHEA is feasible and safe for anastomosis after laparoscopic left hemicolectomy. The KHEA technique could reduce operation time and hospital costs with complication rates comparable to stapling.

## Introduction

Laparoscopic colectomy is widely performed for colon cancer (1), but many variations exist for the method of anastomosis after laparoscopic radical resection for cancer (2–4). Left hemicolectomy may sometimes be a complex procedure [mobilization of the splenic flexure, unexpected adhesions or tumor invasion, intraoperative vascular problems (5, 6). Complete laparoscopic colectomy with an intracorporeal reconstruction technique requires advanced surgical skills and may increase operation time, hospitalization costs, and/or the risk of abdominal contamination (7–9).

The recent advent of barbed sutures has made manual suturing more convenient and quicker because of good tissue adhesion and eliminated need for knot tying. At present, the barbed suture is mainly used for gastrointestinal anastomoses (10, 11) and urinary tract surgery (12), but there are very few reports on left colonic anastomosis.

In this study, we describe the details of a novel extracorporeal anastomosis (ECA) with barbed thread—a knotless hand-sewn end-to-end anastomosis (KHEA) technique—with a video and compare the perioperative outcomes of KHEA to those of stapled anastomosis.

## Material and methods

### Patients selection and data

This was an Institutional Review Board approved study. We retrospectively analyzed 226 patients who underwent laparoscopic-assisted left hemicolectomy (resection of the last third of the transverse colon, descending and upper sigmoid colon) from 2010 to 2021 at the General Surgery Department of Ruijin Hospital, Shanghai, China. The clinical data were recorded prospectively in the database of Ruijin Hospital, and the results were evaluated retrospectively. Patients were divided into two groups: KHEA and stapled anastomosis.

The inclusion criteria for this study included (1) age between 18 and 85 years; (2) diagnosis of colonic adenocarcinoma by colonoscopy, computed tomography (CT), and pathological examination; (3) clinical T stage I to IVa without distant metastases; and (4) laparoscopic-assisted left hemicolectomy. Exclusion criteria were (1) multiple primary tumors; (2) other previous or concurrent major abdominal surgery; (3) metastases found during surgery; (4) associated enterostomy; and (5) unavailable or incomplete clinical data.

**Figure 1:**
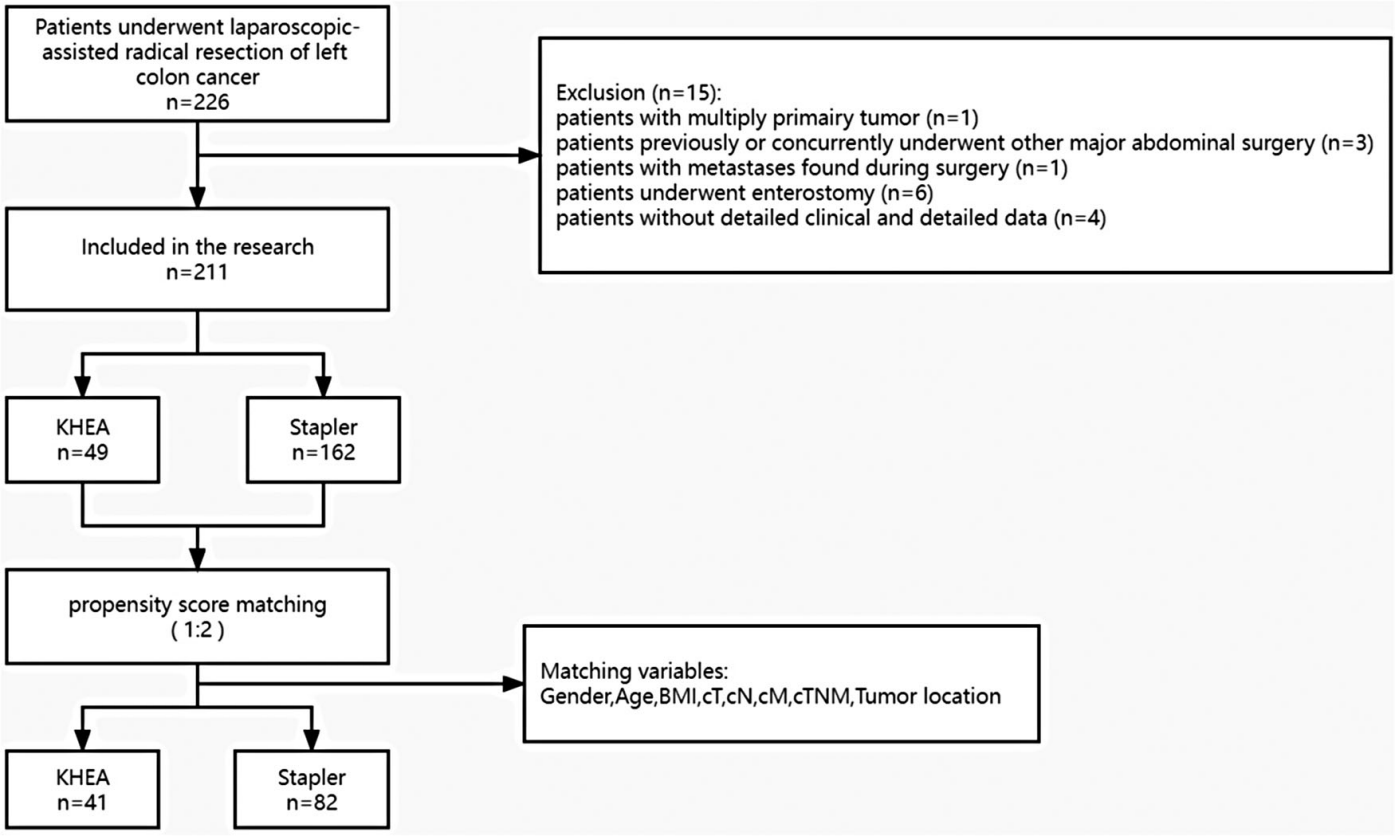
Flow chart.

A total of 211 patients were included in the study: 49 underwent the KHEA technique and 162 underwent stapled anastomosis. A 1:2 propensity score matching (PSM) was performed (Figure 1). All surgeries were performed by physicians who had crossed the learning curve of laparoscopic radical colectomy.

Preoperative demographic and clinicopathologic characteristics included age, gender, body mass index (BMI), TNM stage based on the AJCC Staging Manual 8th edition, and tumor location. Operative data included anastomosis complications, anastomotic time, days to bowel function recovery, postoperative hospital stay, and hospital costs. The postoperative complications were classified according to the Clavien–Dindo grading (13). The anastomosis leak was graded by the modified International Study Group of Rectal Cancer (ISREC) classification (14, 15).

### Surgical technique

**Figure 2:**
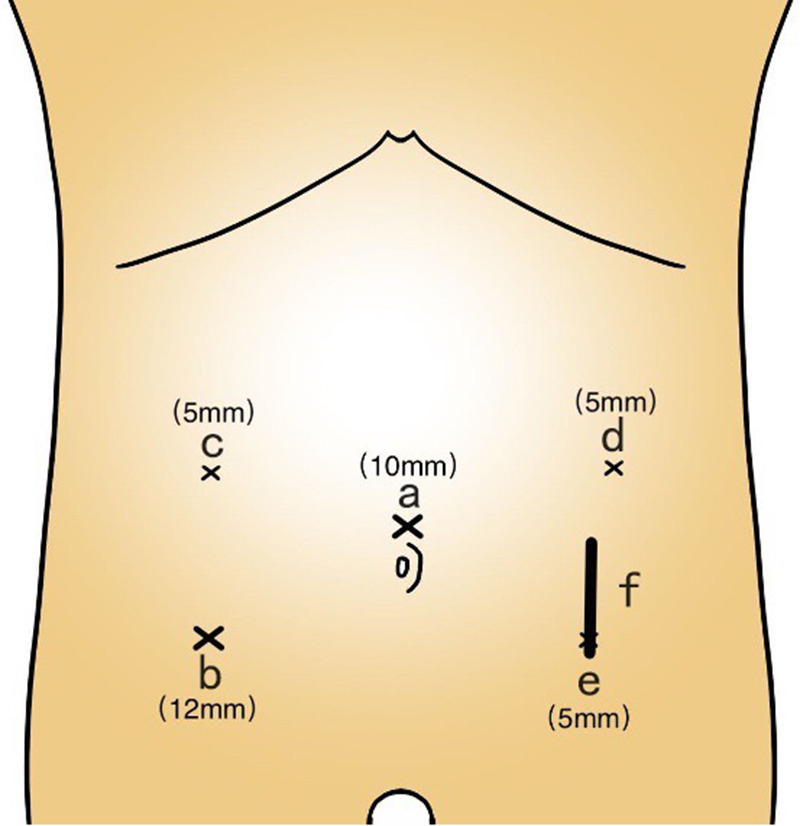
Trocar placement. (**A**) Camera port for laparoscope. (**B**) Manipulation port. (**C–E**) Assistant ports. (**F**) Abdominal incision extended for specimen resection and extraction (approximately 5 cm).

The patient was placed in the supine split-leg position. Under general anesthesia, pneumoperitoneum was established and maintained at 15 mmHg, and five trocar ports were placed (Figure 2). Exploration of the abdominal cavity identified any peritoneal, liver, or other distant metastasis. Surgery was performed according to the complete mesocolic excision principle, with proximal and distal margins of at least 5 cm. For tumors located at the sigmoid-descending colon junction, mobilization of the splenic flexure depends on the tension of the colon.

#### KHEA procedure

The technique consists of two steps: (1) left lower longitudinal incision and (2) continuous double-layer hand-sewing with knotless barbed suture (Supplementary Video).

**Figure 3:**
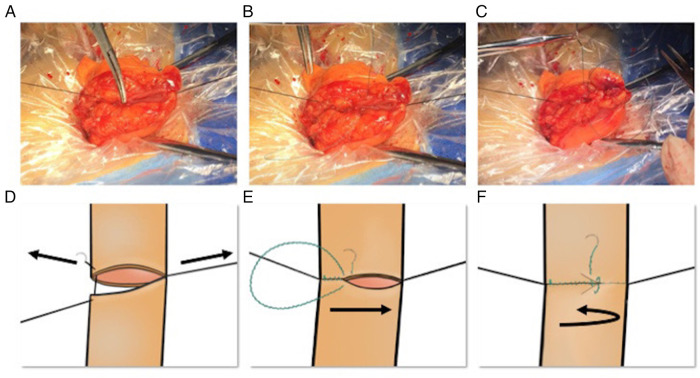
Intraoperative photograph and schematic illustration of KHEA. (**A,D**) Colon suspension: identification of the mesenteric and the mesangial borders of the two colonic extremities, suspended with single-strand sutures. These two threads are used to maintain tension and lift the bowel. (**B,E**) Inner layer suture: We use barbed suture to perform continuous full-thickness suture from the opposite mesangial to the mesenteric border. Suture bites are placed 3–5 mm apart and 2–3 mm from the cut edge of the tissue. (**C,F**) Outer layer reinforcement: We continuously suture the serosa from the mesangial to the mesangial border. Suture bites are placed 5 mm apart and 5 mm from the line of anastomosis. The outer layer suture completely buries the inner suture. KHEA, knotless hand-sewn end-to-end anastomosis.

(1) Abdominal incision: A longitudinal incision was made at the left lower quadrant of the abdomen (approximately 5 cm). The tumor-bearing colon segment was extracted, and the tumor completely resected. The upper and lower resection margins were both over 5 cm.
(2) Colon suspension: The mesenteric border and the opposite mesangial border of the two colon ends were identified and the two extremities were suspended with one single-strand suture each. These two threads were used to maintain tension and lift the bowel (Figure 3A).
(3) Inner layer suture: Barbed suture was then used to perform a continuous full-thickness suture from the mesangial to the mesenteric border. Suture bites were placed 3–5 mm apart and 2–3 mm from the cut edge of the tissue (Figure 3B).
(4) Outer layer suture: The serosa was sutured from the opposite mesangial to the mesenteric border in a continuous fashion. Suture bites were placed 5 mm apart and 5 mm from the line of anastomosis. The outer layer completely buried the inner suture line (Figure 3C). Finally, the barbed suture ends were cut as short as possible.

The opposite hemi-circumference of anastomosis is sutured following the same procedures outlined in (2)–(4). Duration of anastomosis was measured from resection of the tumor until completion of the anastomosis.

#### Procedure of stapled anastomosis

A longitudinal incision was made at the mid or left lower abdomen (approximately 5 cm). The tumor-bearing colonic segment was extracted and resected with its mesentery. The proximal and distal resection margins were both at least 5 cm. Both side-to-side linear stapler and end-to-side circular stapler anastomoses were included in this study. Duration of anastomosis was measured from resection of tumor until completion of the anastomosis.

Linear stapler anastomosis procedure: A linear stapler is inserted into both proximal and distal stump and the common opening is closed with a second linear stapler to complete the anastomosis.

Circular stapler anastomosis procedure: The anvil of a circular stapler is inserted into the distal stump and the purse-string was secured. The circular stapler is inserted into the proximal stump, and the common opening is closed with a linear stapler to complete the anastomosis.

### Postoperative management

Early mobilization was encouraged. Patients were allowed fluid intake after bowel function recovery, and a liquid diet was started the next day. All patients had an abdominal drain, which was removed when the volume of drainage was less than 20 ml. Patients with a normal postoperative course were discharged on postoperative day (POD) 8. Verbal and written instructions specifying warning signs were given to all patients. All patients were followed up by clinic visits or phone calls every 3 months for the first year after surgery. When complications were suspected, endoscopy or CT scan was performed.

### Statistical analysis

Statistical analysis was performed using SPSS version 16.0 (IBM Corporation, Chicago, IL, United States). Continuous variables were expressed as mean ± standard deviation. Student's *t*-test was used for independent samples comparison, the chi-square test and Fisher's exact test was used to compare definite variables. A *p* value <0.05 was considered statistically significant. Schematic diagrams of the surgery were drawn using Sketchbook (IpadOS, Autodesk, United States).

## Results

### Demographic and clinicopathologic characteristics

After the 1:2 PSM, 123 participants with similar preoperative characteristics (age, BMI, TNM stage, and tumor location) were enrolled in the study: 41 in the KHEA and 82 in the stapler group (Table 1).

**Table 1:** Demographic and clinicopathologic characteristics.

Characteristic		Before matching			After matching		
		KHEA	Stapler	*p*	KHEA	Stapler	*p*
		*N* = 49	*N* = 162		*N* = 41	*N* = 82	
Gender (%)	Male	33 (67.3)	111 (68.5)	0.877^a^	29 (70.7)	53 (64.6)	0.320^a^
	Female	16 (32.7)	51 (31.5)		12 (29.3)	29 (35.4)	
Age, years [mean (SD)]		59.1 (12.613)	63.6 (12.290)	0.026^b^	61.7 (10.900)	61.0 (12.172)	0.742^b^
BMI, kg/m^2^ [mean (SD)]		23.4 (2.929)	23.4 (3.146)	0.949^b^	23.5 (2.768)	23.3 (2.914)	0.700^b^
cT (%)	cT1	13 (26.5)	31 (19.1)	0.463^a^	12 (29.3)	16 (19.5)	0.437^a^
	cT2	5 (10.2)	13 (8.0)		5 (12.2)	6 (7.3)	
	cT3	17 (34.7)	76 (46.9)		15 (36.6)	36 (43.9)	
	cT4	14 (28.6)	42 (25.9)		9 (22.0)	24 (29.3)	
cN (%)	cN0	30 (61.2)	102 (63.0)	0.892^a^	29 (70.7)	47 (57.3)	0.383^a^
	cN1	10 (20.4)	35 (21.6)		7 (17.1)	19 (23.2)	
	cN2	9 (18.4)	25 (15.4)		5 (12.2)	16 (19.5)	
cM (%)	cM0	46 (93.9)	154 (95.1)	0.491^a^	40 (97.6)	75 (91.5)	0.186^a^
	cM1	3 (6.1)	8 (4.9)		1 (2.4)	7 (8.5)	
cTNM (%)	I	17 (34.7)	38 (23.5)	0.303^a^	16 (39.0)	19 (23.2)	0.231^a^
	II	13 (26.5)	63 (38.9)		13 (31.7)	27 (32.9)	
	III	16 (32.7)	53 (32.7)		11 (26.8)	29 (35.4)	
	IV	3 (6.5)	8 (4.9)		1 (2.4)	7 (8.5)	
Tumor location	SDJ	24 (49.0)	48 (29.6)	0.004^a^	19 (46.3)	35 (42.7)	0.487^a^
	DC	22 (44.9)	75 (46.3)		20 (48.8)	37 (45.1)	
	TC	3 (6.1)	39 (24.1)		2 (4.9)	10 (12.2)	

KHEA, knotless hand-sewn end-to-end anastomosis; SD, standard deviation; BMI, body mass index; SDJ, sigmoid-descending colon junction; DC, descending colon; TC, transverse colon.

^a^ Chi-square test.

^b^ Student’s t-test.

### Short-term outcomes and costs

Table 2 shows the comparison of short-term outcomes and costs. There was no statistically significant difference in the mean bowel function recovering day (2.61 vs. 2.71, *p* = 0.466), duration of hospital stay (8.61 vs. 7.91, *p* = 0.407), or rate of postoperative complications (14.6% vs. 11.0%, *p* = 0.563). The occurrence of anastomotic leak, graded by the modified classification of International Study Group of Rectal Cancer (ISREC), was observed in 11 patients: 6 in the stapler group (7.3%) and 5 in the KHEA group (12.2%) (*p* > 0.05). Anastomotic bleeding at the colonic anastomotic site was observed in one patient in the stapler group on POD 2. This complication was treated with local adrenaline injection and endoscopic monopolar electrocautery. No anastomotic stenosis or bowel obstruction was observed in either group. One patient in the stapler group had a wound disruption while another patient in the KHEA group had a surgical site infection: all patients were discharged after local treatment. One patient in the stapler group developed a chyle fistula after operation but was discharged from the hospital after being placed on a fat-free diet and clear drainage fluid was noted. Completion of the anastomosis required a shorter time in the KHEA group (mean 7.8 vs. 11.9, *p* < 0.001) than in the stapler group. The surgery cost was also significantly decreased in the KHEA group (mean 46,569.71 vs. 50,915.35 CNY, *p* < 0.05) for V-Loc vs. stapled anastomosis, respectively.

**Table 2:** Comparison of short-term outcomes and costs.

	KHEA	Stapler	*p*
	*N* = 41	*N* = 82	
Anastomotic complications (%)			
Leakage	5 (12.2)	6 (7.3)	0.439^a^
Grade A	3 (7.3)	2 (2.4)	
Grade B	2 (4.8)	4 (4.8)	
Grade C	0	0	
Hemorrhage	0	1 (1.2)	
Stenosis	0	0	
Other complications (%)			
Bowel obstruction	0	0	
Wound-healing complications	1 (2.4)	1 (2.4)	
Chyle leakage	0	1 (2.4)	
Time to complete anastomosis, min [mean (SD)]	7.85 (1.22)	11.92 (1.28)	0.005^b^
Bowel function recovery days, days (SD)	2.61 (0.74)	2.71 (0.68)	0.466^b^
Postoperative hospital days, days (SD)	8.61 (6.05)	7.91 (3.22)	0.407^b^
Hospitalization cost^c^ (SD)	46,569.71 (10,415.15)	50,915.38 (7,248.56)	0.008^b^

KHEA, knotless hand-sewn end-to-end anastomosis; SD, standard deviation.

^a^ Fisher's exact test.

^b^ Student’s t-test.

^c^ Chinese Yuan.

## Discussion

In this study, the mean duration for completion of anastomosis and operation costs were statistically significantly decreased in the KHEA group, while no statistically significant difference was found in the rate of postoperative complications, delay to bowel function recovery, or duration of hospital stay.

The minimally invasive approach for colectomy can be performed *via* either a “minimally invasive assisted” technique with ECA or a “total minimally invasive” technique with intracorporeal anastomosis (ICA), i.e., performing the anastomosis in the abdominal cavity under the direct view of a laparoscope. In recent years, ICA has received increasing focus, but its superiority over extracorporeal anastomosis is still inconclusive (16). Several studies have shown that that ICA has advantages over ECA such as shorter length of incision, less estimated blood loss, and shorter time to bowel function recovery (2, 17, 18). However, two recent high-quality randomized controlled trials (8, 19) and one study on robotic left colectomy (20) failed to show that outcomes after ICA were better than after ECA. The advantages of the KHEA technique could be a further argument in favor of the ICA technique.

For left hemicolectomy, anastomosis can be performed either manually or with staplers. As reported, no statistically significant superiority has been found between these two methods with respect to safety or anastomotic leakage (4, 21). Based on our experience, an end-to-end hand-sewn anastomosis could save approximately 3–5 cm of colon segment compared with stapled anastomosis, enabling surgeons to avoid unnecessary mobilization of the colon and the longitude tension of anastomosis. However, the traditional hand-sewing anastomosis technique requires higher technical skills from surgeons and takes a longer operation time than stapled anastomosis. In our experience, the hand-sewn technique using the new KHEA technique facilitates an easy anastomosis with comparable outcomes.

We extract the bowel specimen from a left lower quadrant incision, approximately 5 cm long. Compared with the midline incision, the left lower incision allows for extraction of the colon segment with less tension, thus avoiding unnecessary bowel dissection and incision extension and may cause less pain for patients. Also, a midline incision could lead to higher morbidity and, particularly, incisional hernia as reported in a systemic review (22).

In recent years, the use of barbed suture has proven to be safe and efficient in urinary tract surgery (23), gynecological surgery (24), and gastrointestinal anastomosis (10, 11). Although multifilament absorbable suture was generally used for gastrointestinal anastomosis, since the application of barbed sutures, more and more surgeons prefer the knotless technique because of the lower operational difficulty, especially in total minimally invasive operations. Applying barbed suture in anastomosis can reduce the number of knots and results in less operation time.

In our series, the postoperative complications were comparable between KHEA and stapled anastomosis. As tension on the anastomosis and poor vascularization of the proximal colon limb are among the risk factors for anastomotic leakage (25), we believe that the KHEA technique might decrease the leakage rate. We found that less mobilization of the bowel is required for the KHEA technique, which might potentially reduce the risk of marginal vascular injury such as injury to Riolan's arch. Moreover, manual suturing may potentially reduce the incidence of anastomotic bleeding (26) because of a better view of the bowel mucosa.

Based on our experience, special attention should be paid to the cut end of the barbed suture material. We recommend cutting the end flush against the intestinal wall to prevent adherence to surrounding tissues, which are a potential source of intestinal obstruction.

We recognize certain limitations in our study. First, this was not a randomized controlled study. We performed propensity score matching to minimize the effects of potential biases due to preoperative patient characteristics. Second, all the health economics calculations were based on the Chinese medical system and might vary in different countries with different insurance policies. Third, we did not evaluate the long-term outcomes. Late-onset postoperative complications such as incisional hernias were not evaluated.

## Conclusion

The KHEA technique is a safe, economical, convenient, and feasible anastomosis method for laparoscopic left hemicolectomy. This technique could substantially reduce hospitalization cost and operational time with comparable complication rates. It may also have more potential benefits because of the less range of bowel dissection and shorter abdomen incision.

## Data Availability

The raw data supporting the conclusions of this article will be made available by the authors, without undue reservation.

## References

[1] Lacy A. Colon cancer: laparoscopic resection. Ann Oncol. (2005) 16:ii88–92. 10.1093/annonc/mdi733 15958483

[2] Carnuccio P Jimeno J Parés D. Laparoscopic right colectomy: a systematic review and meta-analysis of observational studies comparing two types of anastomosis. Tech Coloproctol. (2014) 18(1):5–12. 10.1007/s10151-013-1029-4 23686680

[3] Selvy M Mattevi C Slim K Pezet D Pereira B Le Roy B. Intra-versus extracorporeal anastomosis in laparoscopic right colectomy: a meta-analysis of 3699 patients. Int J Colorectal Dis. (2020) 35(9):1673–80. 10.1007/s00384-020-03675-y 32691134

[4] Matos D Atallah ÁN Castro AA Silva Lustosa SA. Stapled versus handsewn methods for colorectal anastomosis surgery. Cochrane Database Syst Rev. (2001) (3):CD003144. 10.1002/14651858.CD003144 11687041

[5] Wang Y Shu W Ouyang A Wang L Sun Y Liu G. The new concept of physiological “Riolan’s Arch” and the reconstruction mechanism of pathological Riolan’s arch after high ligation of the Inferior mesenteric artery by CT angiography-based small vessel imaging. Front Physiol. (2021) 12:641290. 10.3389/fphys.2021.641290 34239446 PMC8257958

[6] Titu LV Tweedle E Rooney PS. High tie of the inferior mesenteric artery in curative surgery for left colonic and rectal cancers: a systematic review. Dig Surg. (2008) 25(2):148–57. 10.1159/000128172 18446037

[7] Ohmura Y Suzuki H Kotani K Teramoto A. Intracorporeal hemi-hand-sewn technique for end-to-end anastomosis in laparoscopic left-side colectomy. Surg Endosc. (2020) 34(9):4200–5. 10.1007/s00464-020-07612-6 32399939

[8] Dohrn N Yikilmaz H Laursen M Khesrawi F Clausen FB Sørensen F Intracorporeal versus extracorporeal anastomosis in robotic right colectomy: a multicenter, triple-blind, randomized clinical trial. Ann Surg. (2021) 276(5):e294–301. 10.1097/SLA.0000000000005254 35129520

[9] Emile SH. Intracorporeal *versus* extracorporeal anastomosis in laparoscopic right colectomy; earlier recovery, less complications, and more. Br J Surg. (2020) 107(5):614. 10.1002/bjs.11519 32187679

[10] Nemecek E Negrin L Beran C Nemecek R Hollinsky C. The application of the V-loc closure device for gastrointestinal sutures: a preliminary study. Surg Endosc. (2013) 27(10):3830–4. 10.1007/s00464-013-2982-8 23644839

[11] Costantino F Dente M Perrin P Sarhan FA Keller P. Barbed unidirectional V-loc 180 suture in laparoscopic Roux-en-Y gastric bypass: a study comparing unidirectional barbed monofilament and multifilament absorbable suture. Surg Endosc. (2013) 27(10):3846–51. 10.1007/s00464-013-2993-5 23722892

[12] Tewari AK Srivastava A Sooriakumaran P Slevin A Grover S Waldman O Use of a novel absorbable barbed plastic surgical suture enables a “self-cinching” technique of vesicourethral anastomosis during robot-assisted prostatectomy and improves anastomotic times. J Endourol. (2010) 24(10):1645–50. 10.1089/end.2010.0316 20818988

[13] Dindo D Demartines N Clavien PA. Classification of surgical complications: a new proposal with evaluation in a cohort of 6336 patients and results of a survey. Ann Surg. (2004) 240(2):205–13. 10.1097/01.sla.0000133083.54934.ae 15273542 PMC1360123

[14] Rahbari NN Weitz J Hohenberger W Heald RJ Moran B Ulrich A Definition and grading of anastomotic leakage following anterior resection of the rectum: a proposal by the international study group of rectal cancer. Surgery. (2010) 147(3):339–51. 10.1016/j.surg.2009.10.012 20004450

[15] Tzu-Liang Chen W Fingerhut A. Minimal access surgery has its place in the treatment of anastomotic leakage after anterior resection: suggestion for a modification of the International Study Group of Rectal Cancer (ISREC) classification. Surgery. (2021) 170(1):345–6. 10.1016/j.surg.2021.02.044 33781586

[16] Vignali A Elmore U Aleotti F Roberto D Parise P Rosati R. Re-laparoscopy in the treatment of anastomotic leak following laparoscopic right colectomy with intracorporeal anastomosis. Surg Endosc. (2021) 35(11):6173–8. 10.1007/s00464-020-08113-2 33104916

[17] Emile SH Elfeki H Shalaby M Sakr A Bassuni M Christensen P Intracorporeal versus extracorporeal anastomosis in minimally invasive right colectomy: an updated systematic review and meta-analysis. Tech Coloproctology. (2019) 23(11):1023–35. 10.1007/s10151-019-02079-7 31646396

[18] van Oostendorp S Elfrink A Borstlap W Schoonmade L Sietses C Meijerink J Intracorporeal versus extracorporeal anastomosis in right hemicolectomy: a systematic review and meta-analysis. Surg Endosc. (2017) 31(1):64–77. 10.1007/s00464-016-4982-y 27287905 PMC5216072

[19] Allaix ME Degiuli M Bonino MA Arezzo A Mistrangelo M Passera R Intracorporeal or extracorporeal ileocolic anastomosis after laparoscopic right colectomy: a double-blinded randomized controlled trial. Ann Surg. (2019) 270(5):762–7. 10.1097/SLA.0000000000003519 31592811

[20] Achilli P Perry W Grass F Abd El Aziz MA Kelley SR Larson DW Completely intracorporeal anastomosis in robotic left colonic and rectal surgery: technique and 30-day outcomes. Update Surg. (2021) 73(6):2137–43. 10.1007/s13304-021-01061-z 33993462

[21] Slieker JC Daams F Mulder IM Jeekel J Lange JF. Systematic review of the technique of colorectal anastomosis. JAMA Surg. (2013) 148(2):190. 10.1001/2013.jamasurg.33 23426599

[22] Lee L Abou-Khalil M Liberman S Boutros M Fried GM Feldman LS. Incidence of incisional hernia in the specimen extraction site for laparoscopic colorectal surgery: systematic review and meta-analysis. Surg Endosc. (2017) 31(12):5083–93. 10.1007/s00464-017-5573-2 28444496

[23] Pham KN Sack BS O’Connor RC Guralnick ML Langenstroer P See WA V-Loc urethro-intestinal anastomosis during robotic cystectomy with orthotopic urinary diversion. Can Urol Assoc J. (2013) 7(11–12):663. 10.5489/cuaj.508 PMC3840523 24282453

[24] Cong L Li C Wei B Zhan L Wang W Xu Y. V-Loc™ 180 suture in total laparoscopic hysterectomy: a retrospective study comparing polysorb to barbed suture used for vaginal cuff closure. Eur J Obstet Gynecol Reprod Biol. (2016) 207:18–22. 10.1016/j.ejogrb.2016.09.012 27816737

[25] Morse BC Simpson JP Jones YR Johnson BL Knott BM Kotrady JA. Determination of independent predictive factors for anastomotic leak: analysis of 682 intestinal anastomoses. Am J Surg. (2013) 206(6):950–6. 10.1016/j.amjsurg.2013.07.017 24070663

[26] Golda T Zerpa C Kreisler E Trenti L Biondo S. Incidence and management of anastomotic bleeding after ileocolic anastomosis. Colorectal Dis. (2013) 15(10):1301–8. 10.1111/codi.12309 23710632

